# Microbial Community Dynamics in Mother’s Milk and Infant’s Mouth and Gut in Moderately Preterm Infants

**DOI:** 10.3389/fmicb.2018.02512

**Published:** 2018-10-22

**Authors:** Elena Biagi, Arianna Aceti, Sara Quercia, Isadora Beghetti, Simone Rampelli, Silvia Turroni, Matteo Soverini, Angelo Vittorio Zambrini, Giacomo Faldella, Marco Candela, Luigi Corvaglia, Patrizia Brigidi

**Affiliations:** ^1^Unit of Microbial Ecology of Health, Department of Pharmacy and Biotechnology, University of Bologna, Bologna, Italy; ^2^Neonatal Intensive Care Unit, Department of Medical and Surgical Sciences, University of Bologna, Bologna, Italy; ^3^Department of Quality, Innovation, Safety, Environment, Granarolo S.p.A., Bologna, Italy

**Keywords:** infant gut microbiota, infant oral microbiota, milk microbiota, microbiota assembly, breastfeeding, latching, moderately preterm infants

## Abstract

Mother’s own milk represents the optimal source for preterm infant nutrition, as it promotes immune defenses and gastrointestinal function, protects against necrotizing enterocolitis, improves long-term clinical outcome and is hypothesized to drive gut microbiota assembly. Preterm infants at birth usually do not receive their mother’s milk directly from the breast, because active suckling and coordination between suckling, swallowing and breathing do not develop until 32–34 weeks gestational age, but actual breastfeeding is usually possible as they grow older. Here, we enrolled moderately preterm infants (gestational age 32–34 weeks) to longitudinally characterize mothers’ milk and infants’ gut and oral microbiomes, up to more than 200 days after birth, through 16S rRNA sequencing. This peculiar population offers the chance to disentangle the differential contribution of human milk feeding *per se* vs. actual breastfeeding in the development of infant microbiomes, that have both been acknowledged as crucial contributors to short and long-term infant health status. In this cohort, the milk microbiome composition seemed to change following the infant’s latching to the mother’s breast, shifting toward a more diverse microbial community dominated by typical oral microbes, i.e., *Streptococcus* and *Rothia.* Even if all infants in the present study were fed human milk, features typical of healthy, full term, exclusively breastfed infants, i.e., high percentages of *Bifidobacterium* and low abundances of *Pseudomonas* in fecal and oral samples, respectively, were detected in samples taken after actual breastfeeding started. These findings underline the importance of encouraging not only human milk feeding, but also an early start of actual breastfeeding in preterm infants, since the infant’s latching to the mother’s breast might constitute an independent factor helping the health-promoting assembly of the infant gut microbiome.

## Introduction

The microbial community that colonizes the neonatal body in early life has been acknowledged as one of the main contributors to short and long-term infant health status (Milani et al., 2017), and thus included among key participants in the Developmental Origins of Health and Disease (DOHaD) (Stiemsma and Michels, 2018). The DOHaD hypothesis proposes that environmental exposures during critical time windows in early life can impact on later health and disease by altering fetal and infant programming (Waterland and Michels, 2007). Specifically, it has been demonstrated that early life alterations in the progression of events that bring to the assembly of a healthy-like gut microbiome can predispose to several non-communicable diseases in infancy and childhood, such as necrotizing enterocolitis, asthma and atopic disease, obesity, and neurodevelopmental disorders (Stiemsma and Michels, 2018).

The establishment of the infant gut microbial community is a topic of great interest for both scientific and clinical communities. The impact of many covariates on the composition and establishment of the infant microbiome has been thoroughly explored, pointing out that gestational age, mode of delivery, and type of nutrition [mother’s own milk (MOM), donor’s milk (DHM) or formula] could be among the variables with the greatest impact (Gregory et al., 2016; Chu et al., 2017; Cong et al., 2017; Grier et al., 2017; Hill et al., 2017; Timmerman et al., 2017; Wampach et al., 2017; Dahl et al., 2018). Among these factors, the bacterial community of the mother’s milk is thought to be a relevant player (Pannaraj et al., 2017). The origins of the human milk microbiota are still debated, with hypotheses that take into account a theoretical entero-mammary pathway of translocation together with a bi-directional bacterial transfer between the breast skin and the infant’s mouth during suckling (Ramsay et al., 2004; Fernández et al., 2013; Jeurink et al., 2013; Biagi et al., 2017). In this perspective, it appears that actual breastfeeding, with the infant’s latching to the mother’s breast, might contribute actively to the establishment of the infant gut microbiome, possibly involving bacterial contamination from maternal skin and areola, as well as from the infant’s mouth (Pannaraj et al., 2017). More recently, evidences showing the importance of the social and physical environment surrounding the mother-infant dyad (e.g., how many people take care of the infant and how frequently) in determining the milk microbiota composition have been provided (Meehan et al., 2018). Taken together, these findings suggest the existence of a complex – and still partially unexplored – interconnection between maternal, infant and environmental microbiomes in the establishment of infant microbiomes.

Preterm infants at birth usually do not receive their mother’s milk directly from the breast, because active suckling and coordination between suckling, swallowing and breathing are not developed until 32–34 weeks postmenstrual age (Mizuno and Ueda, 2003). However, with adequate support, effective latching and actual breastfeeding are usually possible as the infants develop and grow older (Academy of Breastfeeding Medicine, 2011). In order to disentangle the differential contribution of human milk feeding *per se* vs. latching and actual breastfeeding in the development of the infant gut microbiota, moderately preterm infants (gestational age 32–34 weeks) were recruited in the present study, and mothers’ milk and infants’ gut and oral microbiomes were longitudinally characterized, before and after the beginning of actual breastfeeding, up to 210 days after birth.

## Materials and Methods

### Subjects Recruitment

Sixteen mother-infant pairs (16 mothers and 21 infants) were recruited at the Neonatal Intensive Care Unit of S. Orsola-Malpighi Hospital in Bologna, Italy. Among the recruited infants, there were 11 singletons and 5 twin pairs (1 monochorionic monoamniotic and 4 monochorionic biamniotic). Infants were recruited if moderately preterm (gestational age 32–34 weeks, according to the definition of the World Health Organization^1^) and receiving any human milk feeding (MOM and/or DHM). Exclusive formula feeding was considered as an exclusion criterion. Demographic and clinical data were recorded in a specific case report form and summarized in Table 1. MOM samples, infant stools and oral swabs were collected at least at birth and 4, 7, 14, 21, and 30 days after birth. Whenever possible more samples were collected later on, up to 210 days after birth (Supplementary Figures S1–S3). Stools were collected from diapers using a standard sterile collection tube. Collection of all MOM samples was performed with the aid of a breast pump connected to sterile disposable collection kits into sterile *ad hoc* containers, in accordance with the guidelines for human milk collection used in human milk banks (Committee on Nutrition - Section on Breastfeeding - Committee on Fetus, 2017). Oral samples were obtained by gently swabbing a sterile cotton-tipped applicator on the inside of the infant’s cheek. All samples were immediately delivered to the laboratory of the Unit of Microbial Ecology of Health, Department of Pharmacy and Biotechnology, University of Bologna (Bologna, Italy) and stored at -80°C until analysis. All mothers signed a written consent form. The study was approved by the Ethics Committee of the S. Orsola Malpighi Hospital in Bologna (study protocol 53/2014/U/Tess) and was conducted according to the principles expressed in the Declaration of Helsinki. Methods were carried out in accordance with the approved guidelines.

**Table 1:** Anthropometric and clinical features of the mother-infant pairs (or triads) recruited.

Anthropometric/clinical feature	ratio (%) or mean ± SD
Gestational age at delivery	33.4 ± 1.0
Weight at birth (g)	1787.3 ± 444.9
Female and male	13/21 (61.9%) and 8/21 (38.1%)
Vaginal delivery	2/21 (9.5%)
C-section	19/21 (90.4%)
Twins	10/21 (47.6%)
Sepsis	4/21 (19.0%)
NEC	2/21 (9.5%)
Probiotic administration (*Lactobacillus reuteri* DSM 17938)	17/21 (80.9%)
Surfactant administration	3/21 (14.2%)
Antibiotic administration	14/21 (66.6%)
Antimycotic administration (fluconazole)	1/21 (4.8%)
Enteral feeding	21/21 (100%)
Breastfeeding	15/21 (71.4%)

*Descriptive features, including drug therapy, probiotics administration and feeding strategy, are reported for each enrolled infant (21 infants and 16 mothers). Data are reported as ratio and percentage, or mean ± SD.*

### Total Bacterial DNA Extraction

Total bacterial DNA was extracted from feces using the DNeasy Blood & Tissue kit (QIAGEN, Hilden, Germany) with a modified protocol (Yu and Morrison, 2004). Briefly, 250 mg of stool samples were resuspended in 1 ml of lysis buffer (500 mM NaCl, 50 mM Tris–HCl pH 8, 50 mM EDTA and 4% SDS) and treated with three bead-beating steps in a FastPrep instrument (MP Biomedicals, Irvine, CA, United States) at 5.5 movements per second for 1 min and kept in ice between treatments. Four 3-mm glass beads and 0.5 g of 0.1-mm zirconia beads (BioSpec Products, Bartlesville, OK, United States) were used for bead-beating. After 15 min of incubation at 95°C, samples were centrifuged at full speed for 5 min at 4°C, then 260 μl of 10 M ammonium acetate were added to the supernatants and the samples incubated for 5 min in ice. After 10 min of centrifugation at full speed at 4°C, the supernatants were collected, and one volume of isopropanol was added. Samples were mixed and incubated in ice for 30 min. DNA was collected by 15 min of centrifugation at full speed at 4°C and the pellet was washed with 70% ethanol. The pellet was then resuspended in 100 μl of TE buffer, and RNA and proteins were removed by treating the samples with 2 μl of DNase-free RNase (10 mg/ml) for 15 min at 37°C and 15 μl of proteinase K at 70°C for 10 min, respectively. DNA was further purified using QIAamp Mini Spin columns (QIAGEN) following the manufacturer’s instructions. For DNA extraction from milk and oral swabs, the same procedures described in Biagi et al. (2017) were followed. Briefly, for milk samples, the same protocol described above for fecal samples was applied to pellets obtained by centrifugation of 2 ml of sample at full speed for 10 min at 4°C. For DNA extraction from oral swabs, the cotton swab was suspended in 500 μl of PBS, vortexed for 1 min and sonicated for 2 min. These last two steps were repeated twice, then two cycles of bead-beating with FastPrep at 5.5 movements per second for 1 min, with 200 mg of 0.5-mm glass beads, were applied. Cotton residues were removed, and the debris was pelleted by centrifugation at 9000 *g* for 5 min. The supernatant was discarded, and the pellet resuspended in 180 μl of enzymatic lysis buffer (QIAGEN). Samples were then treated according to the DNeasy Blood & Tissue kit (QIAGEN) instructions, following the protocol for Gram positive bacteria (Biagi et al., 2017). All extracted DNAs were quantified using the NanoDrop ND-1000 spectrophotometer (NanoDrop Technologies, Wilmington, DE, United States).

### 16S rRNA Gene Amplification and Sequencing

For each sample, the V3–V4 region of the 16S rRNA gene was PCR amplified in 25 μl final volume containing 2.5 μl of microbial DNA (diluted to 5 ng/μl for fecal samples, undiluted for milk and oral swabs), 2X KAPA HiFi HotStart ReadyMix (KAPA Biosystems, Roche, Switzerland), and 200 nM of S-D-Bact-0341-b-S-17/S-D-Bact-0785-a-A-21 primers (Klindworth et al., 2013) carrying Illumina overhang adapter sequences. PCR conditions were set up as follows: initial denaturation at 95°C for 3 min, 25 cycles of denaturation at 95°C for 30 s, annealing at 55°C for 30 s, and extension at 72°C for 30 s, and a final extension step at 72°C for 5 min. PCR amplicons were purified with a magnetic bead-based clean-up system (Agencourt AMPure XP; Beckman Coulter, Brea, CA, United States). Indexed libraries were prepared by limited-cycle PCR using Nextera technology and further cleaned up with AMPure XP magnetic beads (Beckman Coulter). Libraries were pooled at equimolar concentrations (4 nM), denatured with 0.2 N NaOH and diluted to 6 pM before loading onto the MiSeq flow cell. Sequencing on Illumina MiSeq platform was performed by using a 2 × 250 bp paired-end protocol, according to the manufacturer’s instructions (Illumina, San Diego, CA, United States). All samples were analyzed in a unique, dedicated sequencing run. Sequencing reads were deposited in the MG-RAST database^2^.

### Bioinformatics

Data analysis was performed using a pipeline combining PANDAseq (paired-end assembler for Illumina sequences) (Masella et al., 2012) version 2.11 and QIIME (Quantitative Insights Into Microbial Ecology) (Caporaso et al., 2010) version 1.8. High-quality reads were filtered and clustered into operational taxonomic units (OTUs) at a 0.97 similarity threshold using UCLUST (Edgar, 2010) version 1.2.22. Taxonomy was assigned using the RDP (Ribosomal Database Project) classifier version 2.2 against Greengenes database (May 2013 release), and the chimera filtering was performed by discarding all singleton OTUs. Alpha diversity was measured using the observed species and Shannon index metrics. Statistics was performed using RStudio software version 1.0.136 running on R software 3.1.3^3^, implemented with the libraries vegan, gplots, PMCMR, and made4. Filtering of bacterial taxa was performed by keeping the genera and families showing a minimum relative abundance of 0.2% and 1%, respectively, in at least 4% of the considered samples. Beta diversity was estimated by computing Bray-Curtis distances between genus-level relative abundance profiles. Bray-Curtis distances were used for Principal Coordinates Analysis (PCoA), and the significance of separation was tested by permutational multivariate analysis of variance using the function “Adonis” of the vegan package. Bacterial groups with the largest contribution to the ordination space were found by using the function envfit of the R package vegan on the genus relative abundances. As inspired by Stewart et al. (2016, 2017), the variability among genus-level relative abundance microbiota profiles of all available MOM samples was calculated as Euclidean distance matrix and clustering was performed using Ward method. Clustering was visualized using the function “heatmap.2” within the R package gplots and used to group samples into putative Milk Community Types (MCTs). Separation of samples into different MCTs was tested through Bray-Curtis distance PCoA and Adonis test. Wilcoxon test was used to assess significant differences between two groups of samples, whereas Kruskal-Wallis test was used for three or more groups, followed by Tukey and Kramer *post-hoc* test. *P*-values were corrected for multiple comparisons using the Benjamini-Hochberg method. A false discovery rate (FDR) of 5% was used. For comparison, data on the fecal and oral ecosystems of healthy, exclusively breastfed, full term 21-day-old infants, were retrieved from Biagi et al. (2017) and re-analyzed along with the data of the present study.

## Results

Sixteen mother-infant pairs were included in the study. The pre-defined sampling of feces and oral swabs (i.e., at 1, 4, 7, 14, 21, and 30 days after birth) was completed for all infants. For five mother-infant pairs, sampling continued up to 6 or 7 months after birth (Supplementary Figures S1, S2). MOM collection was possible for 13 mothers out of 16, at similar time points as feces and oral swabs (Supplementary Figure S3). The extracted bacterial DNA from a total of 339 samples (138 infants’ fecal samples, 19 of which were meconia, 133 infants’ oral swabs, and 68 MOM samples) was phylogenetically characterized by 16S rRNA gene (V3–V4 region) Illumina sequencing. A mean of 46,786 reads per sample were obtained (range 2,534–504,131); the average number of reads obtained for fecal, oral and milk samples was 46,982, 56,471, and 27,448, respectively.

**FIGURE 1:**
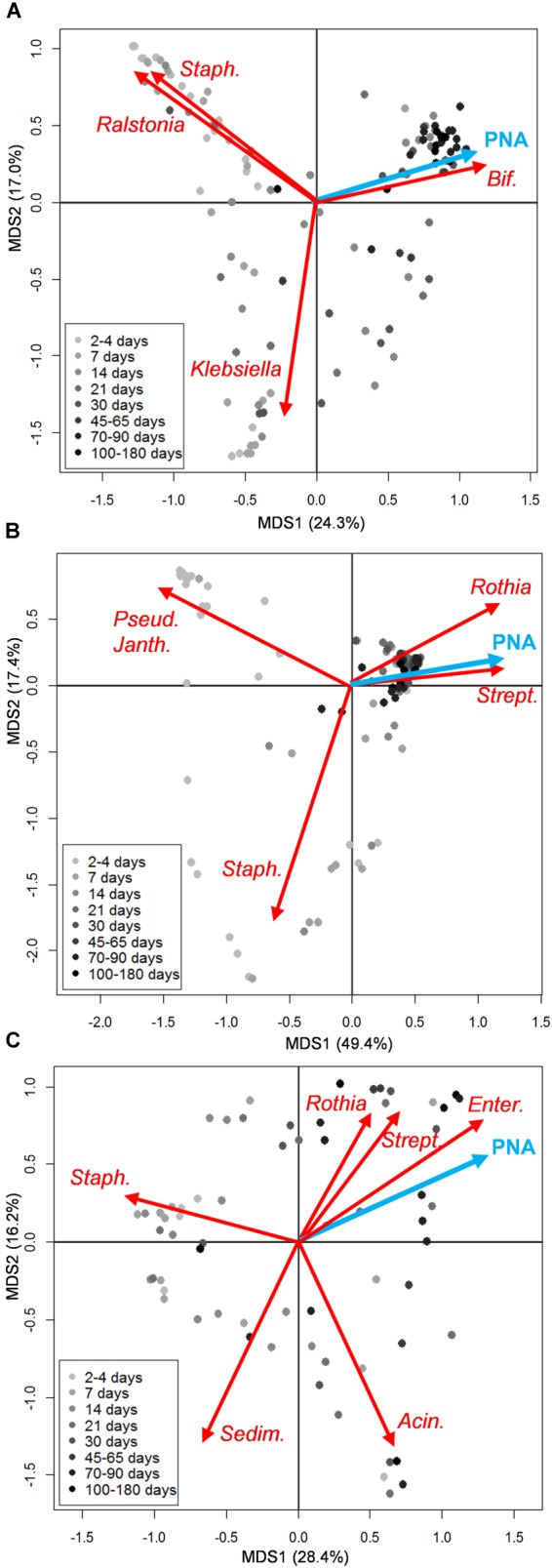
Microbiota structure of the infant’s gut and mouth and mother’s milk in moderately preterm infants, in relation to postnatal age. Principal Coordinates Analyses (PCoA) based on Bray-Curtis distances between genus-level relative abundance profiles of fecal **(A)**, oral **(B)**, and mother’s milk **(C)** samples. Samples are depicted as dots filled in with different shades of gray, from light (earlier samples) to dark (later samples), according to the sampling time (days from birth). A legend for the gray scale is provided. First and second coordination axes are reported in each plot; percentages of variation in the datasets explained by each axis are reported. Postnatal age (PNA, expressed in days from birth at which each sample was taken) as a quantitative environmental variable is depicted as a blue arrow. The biplot of the average bacterial coordinates weighted by the corresponding bacterial relative abundance per sample was superimposed on the PCoA plot for abundant (average relative abundance > 1%) bacterial genera that most significantly contributed to the ordination space (envfit, *P* < 0.001) (red arrows). Some of the genus names are abbreviated as follows: *Staph., Staphylococcus*; *Bif., Bifidobacterium*; *Pseud., Pseudomonas*; *Janth., Janthinobacterium*; *Strept., Streptococcus*; *Enter., Enterococcus*; *Sedim., Sediminibacterium*; *Acin., Acinetobacter*. See Supplementary Figures S1–S3 for details about sampling times for each infant.

According to the Bray-Curtis distance PCoA performed on genus-level microbiota profiles, the structure of both oral and fecal ecosystems tended to converge toward a common configuration over time, whereas higher sample dispersion was noticeable for earlier time points. According to our multivariate analysis, features of the infant microbiota composition which correlated with postnatal age were high abundances of bacteria assigned to the genus *Bifidobacterium* in the case of the fecal ecosystem, and *Streptococcus* and *Rothia* in the oral microbiota (envfit, *P* < 0.001) (Figures 1A,B and Supplementary Figures S4, S5). Such convergent trend was not observed for the milk microbiota (Figure 1C and Supplementary Figure S6). Since 14 out of the 21 enrolled moderately preterm infants received antibiotics within the first week of life, we explored the impact of antibiotic treatment on both fecal and oral microbiota at 7, 14, and 30 days of life, by performing Bray-Curtis distance calculation on genus-level microbiota profiles followed by Adonis test. For what concerns the oral ecosystem, the antibiotic treatment did not show any significant impact on the microbial composition at genus level (Adonis test, *P* > 0.15 and *R*^2^ < 0.08 for data at days 7, 14, and 30). On the contrary, the fecal ecosystem showed a tendency to being impacted by the early administration of antibiotics at days 7 and 14 (Adonis test, *P* = 0.05 and *R*^2^ = 0.11 for data at both days), but this effect was no longer detectable at day 30 (Adonis test, *P* = 0.86 and *R*^2^ = 0.03). However, at day 7 and 14 the large majority of infants taking antibiotics were also hospitalized (100% and 86%, respectively) and not breastfed (86% and 85%, respectively), pointing out that the separate impact of these covariates, i.e., antibiotic administration, breastfeeding and hospitalization, on the microbiota composition of preterm infants could be hard to discriminate.

Meconia were available for 19 out of 21 infants. Confirming previous studies (Hu et al., 2013; Ardissone et al., 2014; Dobbler et al., 2017), the microbiota composition of these samples was highly variable, with no evident relation with the type of birth (vaginal vs. C-section, and break of membranes) (Supplementary Figure S7). However, it should be noted that most samples were from infants born by planned C-section, which makes the calculation of statistical significance of little sense.

**FIGURE 2:**
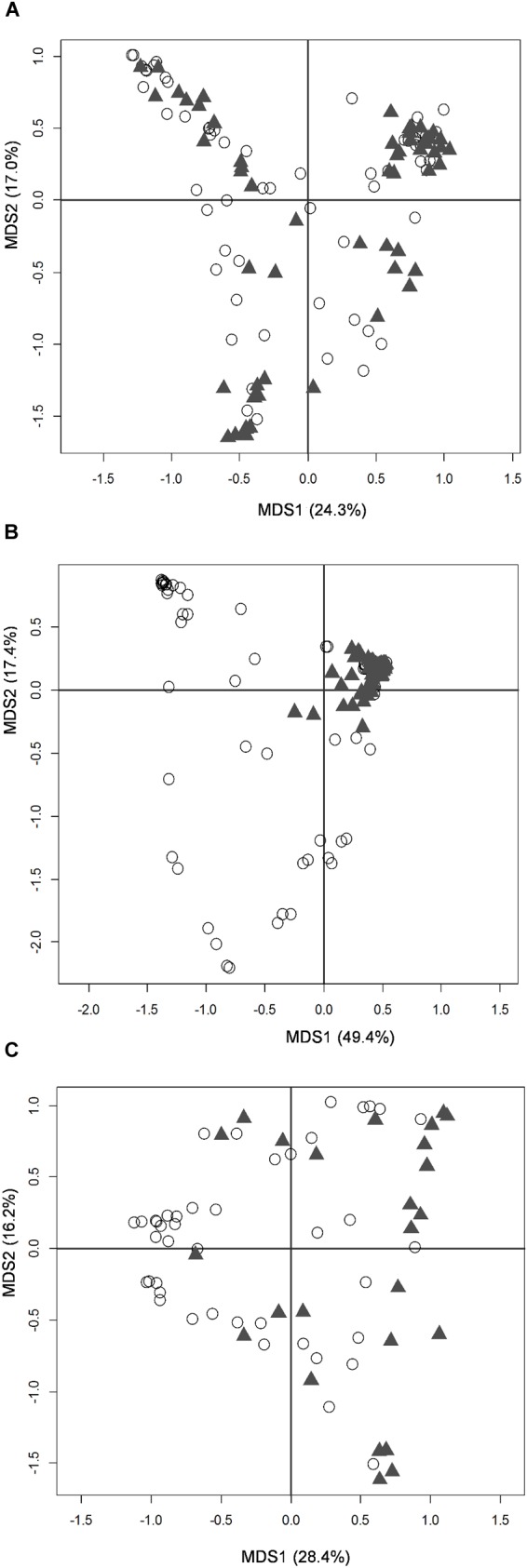
Microbiota structure of the infant’s gut and mouth and mother’s milk in moderately preterm infants, in relation to latching. PCoA based on Bray-Curtis distances between genus-level relative abundance profiles of fecal **(A)**, oral **(B)**, and mother’s milk **(C)** samples. Samples taken before and after latching are depicted as empty dots and filled triangles, respectively. First and second coordination axes, with percentages of plotted variation, are reported as in Figure 1.

The large majority of enrolled infants received exclusive human milk (MOM and/or DHM) or mixed feeding (human milk plus a certain amount of formula); indeed, only 3 fecal samples and 3 oral samples from 2 subjects (twins c13a and c13b) were taken during exclusive formula feeding (Supplementary Figures S1, S2). Neither fecal nor oral samples taken during mixed or exclusive human milk feeding were distinguishable from each other, based on a PCoA of Bray-Curtis distances between genus-level microbiota profiles (Supplementary Figure S8). According to our data, latching and actual breastfeeding had a significant impact on the composition of the infant’s oral microbiota (Figure 2B), as well as on that of the MOM microbiota (Figure 2C) (Adonis test, *P* = 0.001 and *R*^2^ = 0.18 for oral microbiota, *P* = 0.001 and *R*^2^ = 0.10 for MOM microbiota). Conversely, the effect of latching on the infant’s fecal microbiota was not significant (Figure 2A).

**FIGURE 3:**
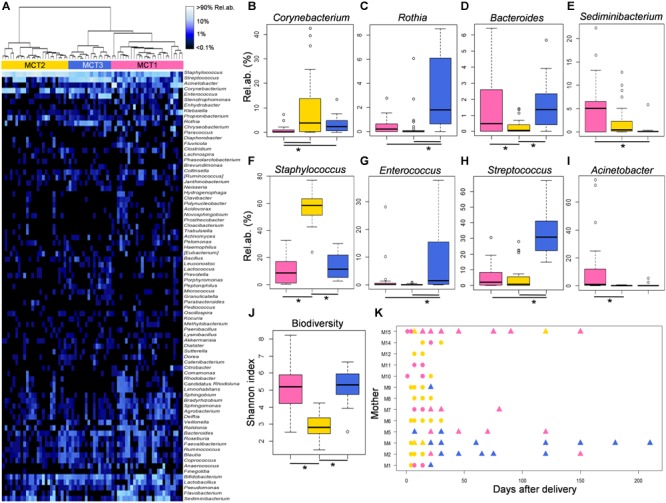
Features of the three Milk Community Types (MCTs). **(A)** MCTs were defined by Ward clustering on Euclidean distances calculated between the genus-level milk microbiota profiles. Samples assigned to MCT1, MCT2, and MCT3 are depicted in pink, gold and blue, respectively. **(B–I)** Relative abundances of bacterial genera for which a significant difference among MCTs was found (Kruskal-Wallis test, FDR corrected *P* < 0.05) are shown as boxplots. **(J)** Levels of alpha diversity calculated as Shannon index are depicted as boxplots for each MCT. Lines and stars below each plot indicate significant differences for pairwise comparisons (*post-hoc* Tukey and Kramer test). **(K)** Dynamics of the milk microbiome through each MCT for each enrolled lactating mother. Symbols represent sampling times for each subject. Triangles indicate samples taken after latching and start of actual breastfeeding.

In order to explore the temporal dynamics of the MOM microbial ecosystem, and their possible associations with changes in the infant’s microbiomes, a “blind” approach (as inspired by Stewart et al., 2016, 2017) was adopted. The variability among genus-level relative abundance microbiota profiles of all available MOM samples was explored by means of a Euclidean distance matrix, which was used to perform clustering analysis. According to the clustering dendrogram, milk samples were separated into three different groups that we defined as putative “Milk Community Types” (MCTs) (Figure 3A). As a confirmation of the samples separation, we carried out a Bray-Curtis distance-based PCoA and verified that samples belonging to different MCTs plotted separately (Adonis test, *P* = 0.001 and *R*^2^ = 0.37) (Supplementary Figure S9). The three suggested MCTs showed significantly different features in terms of microbial composition (Figures 3B–I). In particular, MCT2 samples were characterized by the lowest biodiversity (Kruskal-Wallis test, *P* < 0.0001) (Figure 3J and Supplementary Figure S9) and the highest relative abundance of bacteria assigned to the genus *Staphylococcus* (Kruskal-Wallis test, FDR corrected *P* = 0.005) (Figure 3F). Also, MCT2 seemed to be more associated with samples collected earlier (days after birth, mean ± SD, 16.4 ± 22.9) (Figure 3K), whereas samples taken after the beginning of actual breastfeeding preferentially harbored MCT3 (100% of MCT3 samples were taken after the start of actual breastfeeding) or MCT1 (40% of MCT1 samples were taken after the beginning of actual breastfeeding) (Figure 3K and Supplementary Figure S9). MCT3 was characterized by the highest proportions of bacteria assigned to the genera *Rothia, Enterococcus* and *Streptococcus* (Kruskal-Wallis test, FDR corrected *P* = 0.02, *P* = 0.03, and *P* = 0.01, respectively) (Figures 3C,G,H). Specific features of MCT1 included significantly higher relative abundances of *Sediminibacterium* and *Acinetobacter* (Kruskal-Wallis test, FDR corrected *P* = 0.04 and *P* = 0.02, respectively) (Figures 3E,I).

In order to understand if the MOM microbiota changes over time occurred in parallel to changes in the infants’ oral and gut microbial ecosystems, we explored the composition of fecal and oral samples taken at time points at which the corresponding milk samples were available and clustered into one of the three suggested MCTs. For this analysis, 72 fecal and 76 oral samples were considered; five infants (c3a, c3b, c13a, c13b, and c16) were excluded because their MOM samples were not available. According to a Bray-Curtis PCoA, infant’s fecal samples taken when the MOM harbored a MCT2, mostly including earlier time points (Figure 3K), were significantly different from all the other fecal samples in terms of genus-level microbiota composition (Adonis test, *P* = 0.02 and *R*^2^ = 0.07) (Supplementary Figure S10). Analogously, oral samples taken at time points at which the MOM harbored a MCT2, were significantly different from all the other oral samples (Adonis test, *P* = 0.001 and *R*^2^ = 0.11) and showed higher dispersion on the PCoA space (Supplementary Figure S10). For both feces and oral swabs, the groups of samples corresponding to the three different MCTs included similar percentages of samples taken during mixed or exclusive human milk feeding (percentages of mixed milk in MCT1, MCT2, and MCT3, in fecal samples: 61.5%, 44.4%, and 52.6%; in oral samples: 65.5%, 41.1%, and 52.6%) (Supplementary Figure S10).

Both fecal and oral bacterial communities showed significant compositional differences according to the MCT in which the corresponding milk samples were clustered (Supplementary Figure S11). Indeed, both fecal and oral samples taken in correspondence with milk samples harboring MCT2 showed the highest relative abundance of *Staphylococcus* (Kruskal-Wallis test, FDR corrected *P* = 0.01 and *P* = 0.04, in feces and oral swabs, respectively), a feature that was already detected in the corresponding milk samples (Figure 3F). Conversely, *Rothia* showed the highest relative abundance in both oral and fecal samples taken in correspondence to milk samples harboring MCT3 and the lowest in correspondence to MCT2 (Kruskal-Wallis test, FDR corrected *P* = 0.04 and *P* = 0.01, in feces and oral swabs, respectively), mimicking what already observed in the milk itself (Figure 3C). Interestingly, the sequence reads assigned to the genera *Staphylococcus* and *Rothia* were dominated in all samples by two OTUs (OTU518072 assigned to *Staphylococcus epidermidis*, and OTU537346 assigned to *Rothia mucilaginosa*) that were highly shared among the three types of samples (milk, feces and oral swab) taken from each dyad/triad mother and infant(s). In particular, OTU518072-*S. epidermidis* was detected at relative abundance > 0.1% in at least 1 time point in each of 3 type of samples for 100% of the enrolled infants (Supplementary Figure S12); OTU537346-*R. mucilaginosa* was detected at relative abundance > 0.1% in at least 1 time point in each of 3 type of samples for 56% of the enrolled infants (Supplementary Figure S13).

**FIGURE 4:**
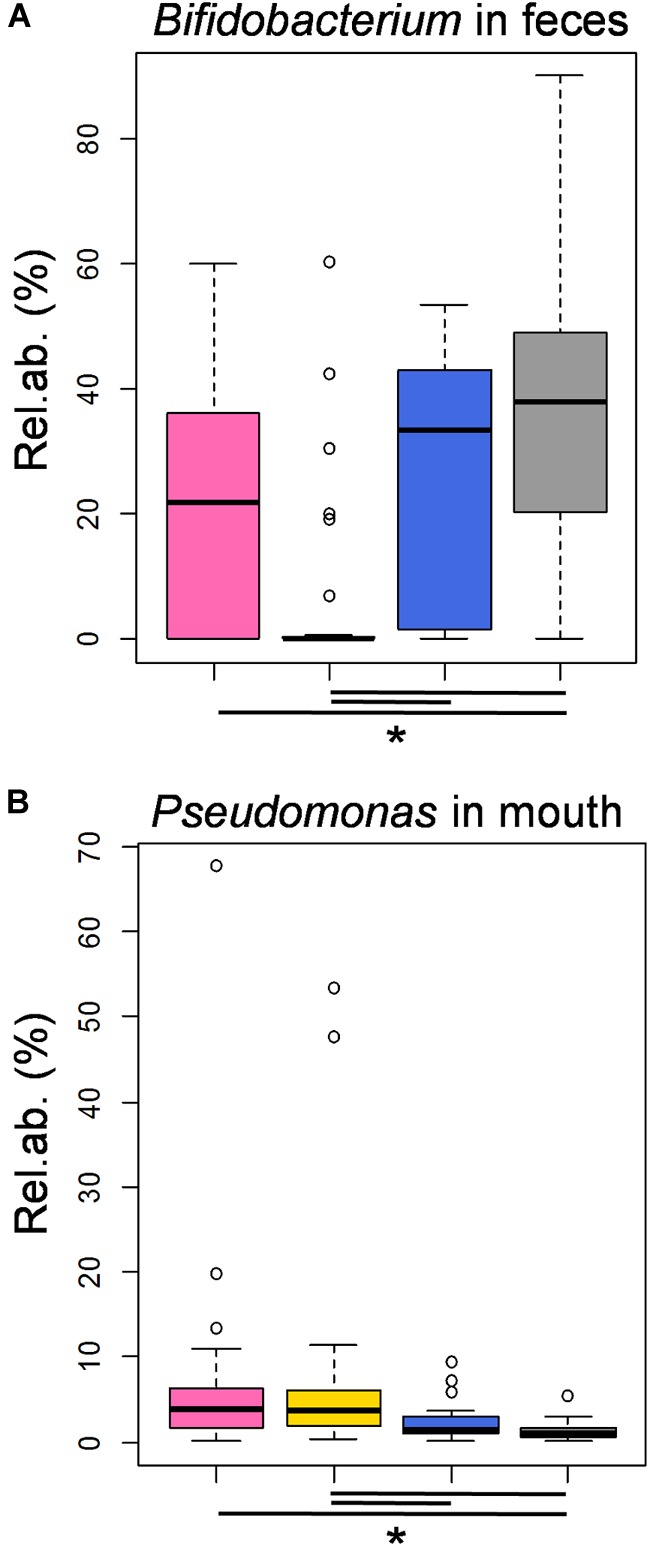
Features of the moderately preterm infants’ fecal and oral bacterial ecosystems associated with the three MCTs, in comparison to healthy, exclusively breastfed, full term infants. Boxplots of the relative abundances of OTUs assigned to the genera *Bifidobacterium* and *Pseudomonas* in fecal **(A)** and oral **(B)** samples, respectively, taken from the enrolled moderately preterm infants at time points in which the corresponding milk samples harbored a MCT1 (pink), MCT2 (gold) or MCT3 (blue). In gray, the data obtained in samples taken from healthy, full term, actively and exclusively breastfed 21-day-old infants (Biagi et al., 2017; sequence reads re-analyzed together with those obtained in the present study). Lines and stars below each plot indicate significant differences for pairwise comparisons (*post-hoc* Tukey and Kramer test, after Kruskal-Wallis test, *P* < 0.05).

Most interestingly, the highest proportion of *Bifidobacterium* in feces and the lowest percentage of *Pseudomonas* in the infant’s mouth were detected in samples taken in correspondence with milk harboring MCT3 (Kruskal-Wallis test, FDR corrected *P* = 0.008 and *P* = 0.02, respectively), which was the MCT most associated with later samples (72.3 ± 63.5 days after birth) and actual breastfeeding (100%) (Figure 3K). It is worth noting that these abundances were comparable to those obtained in fecal and oral samples from healthy, exclusively breastfed, full term 21-day-old infants (from the dataset published in Biagi et al., 2017; Figures 4A,B). According to our analysis at OTU level, a single OTU assigned to *Pseudomonas* spp. was among the most abundant OTUs detected in the whole study, sporadically present at relative abundance between 0.1 and 1% in milk and feces but abundant (>10%) in all oral samples taken during the first 4 days of life (Supplementary Figure S14). Differently, the *Bifidobacterium* genus was composed of a larger variety of OTUs, the most frequently abundant of which were assigned to *Bifidobacterium longum* and *Bifidobacterium breve* (Supplementary Figure S15). These OTUs were detectable in 12 out of 16 meconium samples, then they disappeared or decreased to < 0.1% relative abundance. The time of reappearance of these species of *Bifidobacterium* through the longitudinal sampling performed in this study was highly individual.

## Discussion

Breast milk represents the optimal source of infant nutrition. For preterm infants, exclusive human milk feeding has been linked to improved immune defenses and gastrointestinal function, protection against serious diseases such as necrotizing enterocolitis, and improved long-term clinical outcome (Harding et al., 2017). It is well known that human breast milk harbors its own microbial community that has been hypothesized to be influenced, among other factors such as gestational age, geography and antibiotics, by breastfeeding itself, through bacterial transfer from the infant’s mouth via flow back of milk into the mammary gland during suckling (Pannaraj et al., 2017; Meehan et al., 2018).

In order to examine the specific role of latching and actual breastfeeding in shaping MOM and infant’s oral and gut microbial communities, we included into this study a peculiar population of preterm infants and their mothers. Moderately preterm infants, who are born between 32 and 34 weeks of gestational age, are usually unable to actively suck in the first days/weeks of life (Mizuno and Ueda, 2003), thus initially receiving MOM, DHM and/or formula through an orogastric tube. Mothers of these infants are encouraged to express milk through a breast pump, in order to promote milk production and to guarantee to the infant the benefits related to MOM feeding. Human milk has peculiar nutritional and functional components, which are responsible for improved neurological, immunological, and metabolic outcomes (Schneider and Garcia-Rodenas, 2017). Furthermore, components of human milk are known to contribute to the microbiota assembly along the infant’s gastrointestinal tract (Gregory et al., 2016; Stiemsma and Michels, 2018). With adequate support, as the infants develop and grow older, actual breastfeeding can start. In this peculiar setting, the differential contribution of human milk functional activity vs. latching and actual breastfeeding on the infant’s microbiomes can be observed in a longitudinal monitoring, providing useful information on how feeding directly from the breast can contribute to the preterm infant’s microbiome assembly, in addition to the intrinsic health-promoting effects of milk itself.

In breastfeeding mothers, the composition of the milk microbiome is known to be relatively stable over time (Williams et al., 2017). However, in our cohort, we found that the milk microbiome composition changed following the infant’s latching to the mother’s breast and that it could undergo one or more shifts from a community type to another over time. Indeed, a milk bacterial community with lower diversity and dominated by *Staphylococcus* (the one that we called Milk Community Type (MCT) 2, based on the genus-level microbiota clustering of milk samples) was more frequently retrieved in the first month after delivery and in mothers who did not breastfeed directly. On the contrary, milk samples taken after latching harbored more diverse microbial communities (that we identified as MCT1 and MCT3). In particular, a microbial community dominated by *Streptococcus* and with significantly higher relative abundance of *Rothia* (suggested MCT3), which are both typical oral microbes in children (Jiang et al., 2016; Pettigrew et al., 2016), was associated exclusively with samples taken after latching, sustaining the hypothesis of a retrograde bacterial transfer from the infant’s mouth to the mammary gland during suckling.

The human milk microbiome is hypothesized to seed the infant gut (Pannaraj et al., 2017; Stiemsma and Michels, 2018), but little is known about the importance of the microbial transfer between mother and infant through the act of breastfeeding. Even with all the limitations of a small (21 subjects, sampled longitudinally for a variable time), 16S rRNA gene sequencing-based study, our results showed that different types of microbial communities in milk are associated with different features in the infant’s gut and mouth ecosystems. In particular, the community type that was most strongly associated with actual breastfeeding (MCT3) corresponded to the highest percentages of *Bifidobacterium* in the infant’s gut, with a median relative abundance that was similar to that obtained by our research group in a previous study on healthy, exclusively breastfed, full term infants (Biagi et al., 2017). The gut microbiome of these latter infants can be considered as a reference gold standard, thanks to the restrictive selection criteria used in that study (i.e., healthy, full term infants who were born by vaginal delivery, did not receive any antibiotic, and were exclusively breastfed). The retrieval of high relative abundances of bifidobacteria, i.e., the most important probiotic group residing in the gut of healthy infants (Milani et al., 2017), in moderately preterm infants who were fed directly from the breast, suggests that latching and actual breastfeeding could help shift the gut microbiome of these infants toward the health-associated profile of full term infants. Our study also showed that samples taken before 30 days of life are more likely to be impacted by postnatal administration of antibiotics than later samples. Accordingly, the correlation between MCTs and fecal microbiota might be biased by other covariates linked to the sampling time, such as antibiotic administration and hospitalization, which is frequent in moderately preterm infants during the first week of life.

Observation analogous to those made on the fecal microbiota was made on the oral ecosystem of moderately preterm infants: the lowest relative abundance of *Pseudomonas* was observed in oral samples taken in correspondence to milk samples harboring MCT3, and it was similar to that measured in the mouth of the full term infants from our previous study (Biagi et al., 2017). *Pseudomonas* can be an oral and tracheal pathogen found in preterm infants, particularly associated with intubation practices and antibiotics (Costello et al., 2013; Underwood and Sohn, 2017). It is an interesting finding that *Streptococcus*, a well-known signature of oral ecosystem maturation exerting an inhibitory activity on *Pseudomonas* growth (Hendricks-Muñoz et al., 2015; Whiley et al., 2015), was the dominant genus in milk samples clustered as MCT3, whereas its abundance was significantly lower in both MCT1 and MCT2. Decrease in oral *Pseudomonas* has also been strongly associated with skin-to-skin care in preterm infants (Hendricks-Muñoz et al., 2015). Taken together, our observations strengthen, from a novel, microbiome-centered, point of view, the importance of encouraging not only exclusive human milk feeding, but also an early start of actual breastfeeding in preterm infants, as the infant’s latching to the mother’s breast appears to be an independent factor associated with a health promoting profile of the infant gut microbiome.

## Author Contributions

PB, AZ, GF, and LC conceived the study. AA applied for Ethical Committee approval. SQ, EB, and IB performed the experimental investigation. EB, SR, and MS analyzed bioinformatics and statistics of this work. EB, SQ, and AA wrote the original draft of the manuscript. EB, MC, AA, and ST wrote, reviewed, and edited the manuscript. AA, IB, and SQ collected the samples and curated the data. EB and MS prepared the figures. PB, LC, MC, and EB acquired funding. All authors discussed the results and commented on the manuscript.

## Conflict of Interest Statement

The authors declare that the research was conducted in the absence of any commercial or financial relationships that could be construed as a potential conflict of interest.

## Footnotes

1 http://www.who.int/news-room/fact-sheets/detail/preterm-birth

2 https://www.mg-rast.org/linkin.cgi?project=mgp85715

3 https://www.r-project.org/
