# Supplementary material for: Microbial Community Dynamics in Mother’s Milk and Infant’s Mouth and Gut in Moderately Preterm Infants
Source: Front Microbiol. 2018 Oct 22;9:2512. doi: 10.3389/fmicb.2018.02512 (PMC6204356; doi:10.3389/fmicb.2018.02512)

Supplementary Material

Microbial community dynamics in mother's milk and infant's mouth and gut in moderately preterm infants

Elena Biagi*, Arianna Aceti, Sara Quercia, Isadora Beghetti, Simone Rampelli, Silvia Turroni, Matteo Soverini, Vittorio Zambrini, Giacomo Faldella, Marco Candela, Luigi Corvaglia, Patrizia Brigidi

* Correspondence: Elena Biagi, elena.biagi@unibo.it

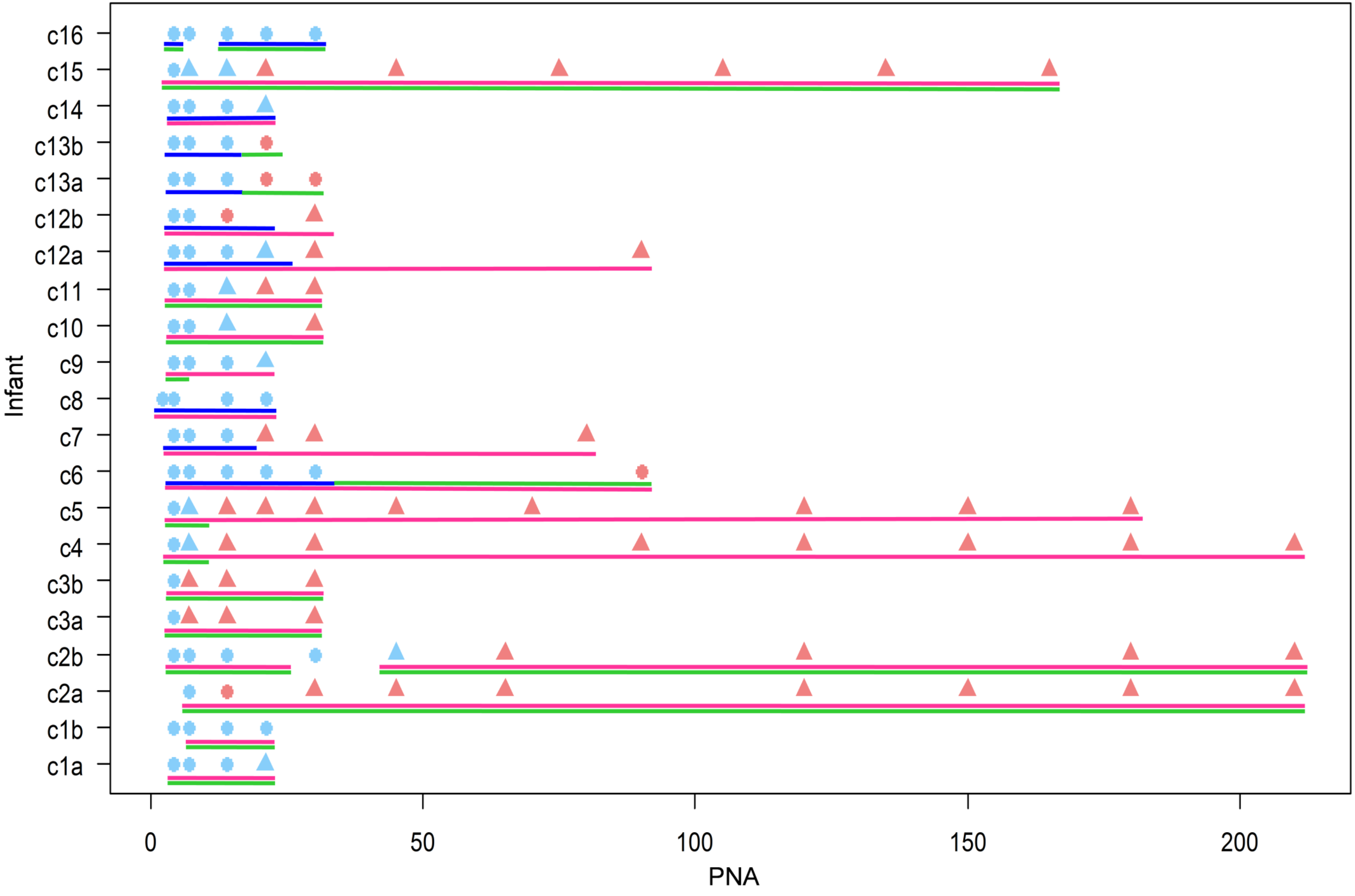

Supplementary Figure S1. Schematic overview of the fecal sampling time for each enrolled infant. Infants are indicated with a progressive number; subjects with the same number and differentiated by «a» and «b» letters are twins. Days of feces collection are plotted as dots or triangles on timelines with postnatal age (PNA) expressed in days. Triangles correspond to fecal samples taken after latching took place. Light blue and light red points correspond to fecal samples taken before and after hospital discharge, respectively. Lines depicted under the sampling points identify the type of feeding (pink, mother's milk; green, infant formula; blue, donor's milk). Feeding line interruptions identify periods in which feeding was suspended because of the occurrence of necrotizing entero-colitis (NEC) (subjects c2b and c16).

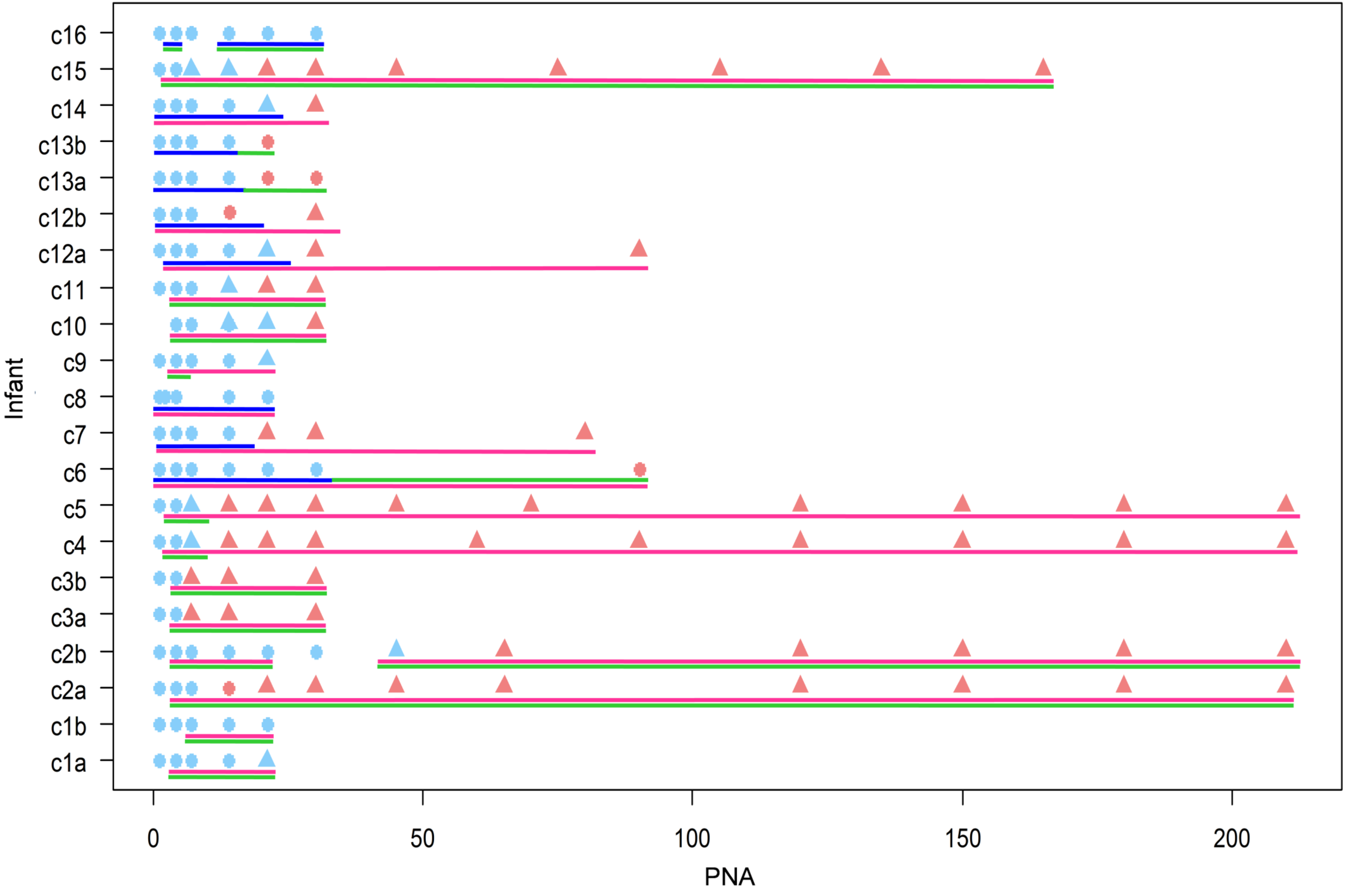

Supplementary Figure S2. Schematic overview of the oral ecosystem sampling time for each enrolled infant. Infants are indicated with a progressive number; subjects with the same number and differentiated by «a» and «b» letters are twins. Days of samples collection are plotted as dots or triangles on timelines with postnatal age (PNA) expressed in days. Triangles correspond to oral samples taken after latching took place. Light blue and light red points correspond to oral samples taken before and after hospital discharge, respectively. Lines depicted under the sampling points identify the type of feeding (pink, mother's milk; green, infant formula; blue, donor's milk). Feeding line interruptions identify periods in which feeding was suspended because of the occurrence of necrotizing entero-colitis (NEC) (subjects c2b and c16).

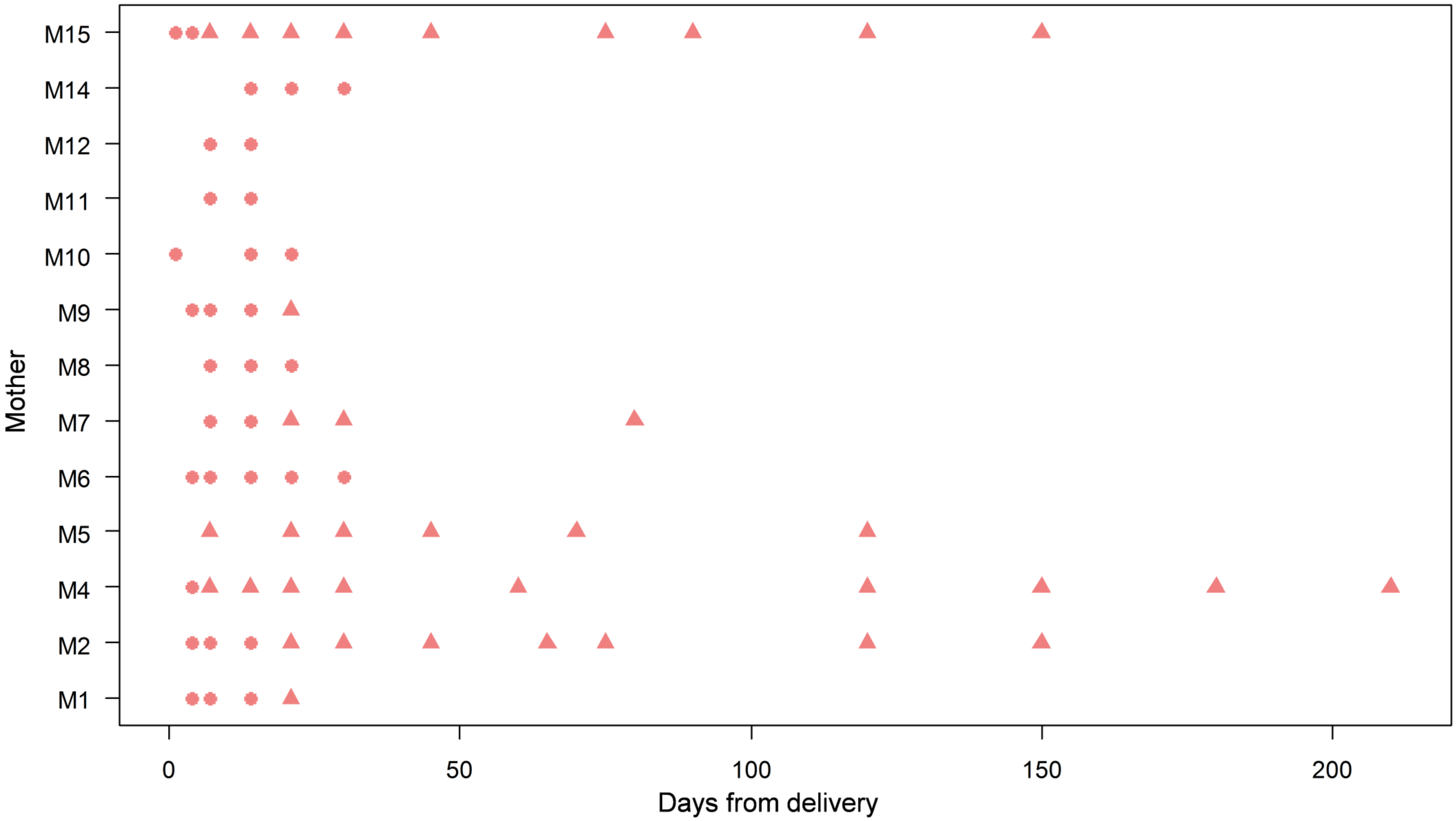

Supplementary Figure S3. Schematic overview of the milk ecosystem sampling time for each enrolled mother. Subjects are indicated with a progressive number. Days of samples collection are plotted as dots or triangles on timelines with distance from delivery expressed in days. Triangles correspond to samples taken after latching took place.

Supplementary Figure S4. Family-level relative abundance profiles of the fecal microbiota of the enrolled 21 infants. Infant codes are reported at the bottom. Infants with the same number and differentiated by «a» and «b» letters are twins. Day of sampling is indicated for each sample. Bacterial families showing a minimum relative abundance of 1% in at least 4% of samples are plotted. Color legend is shown at the bottom.

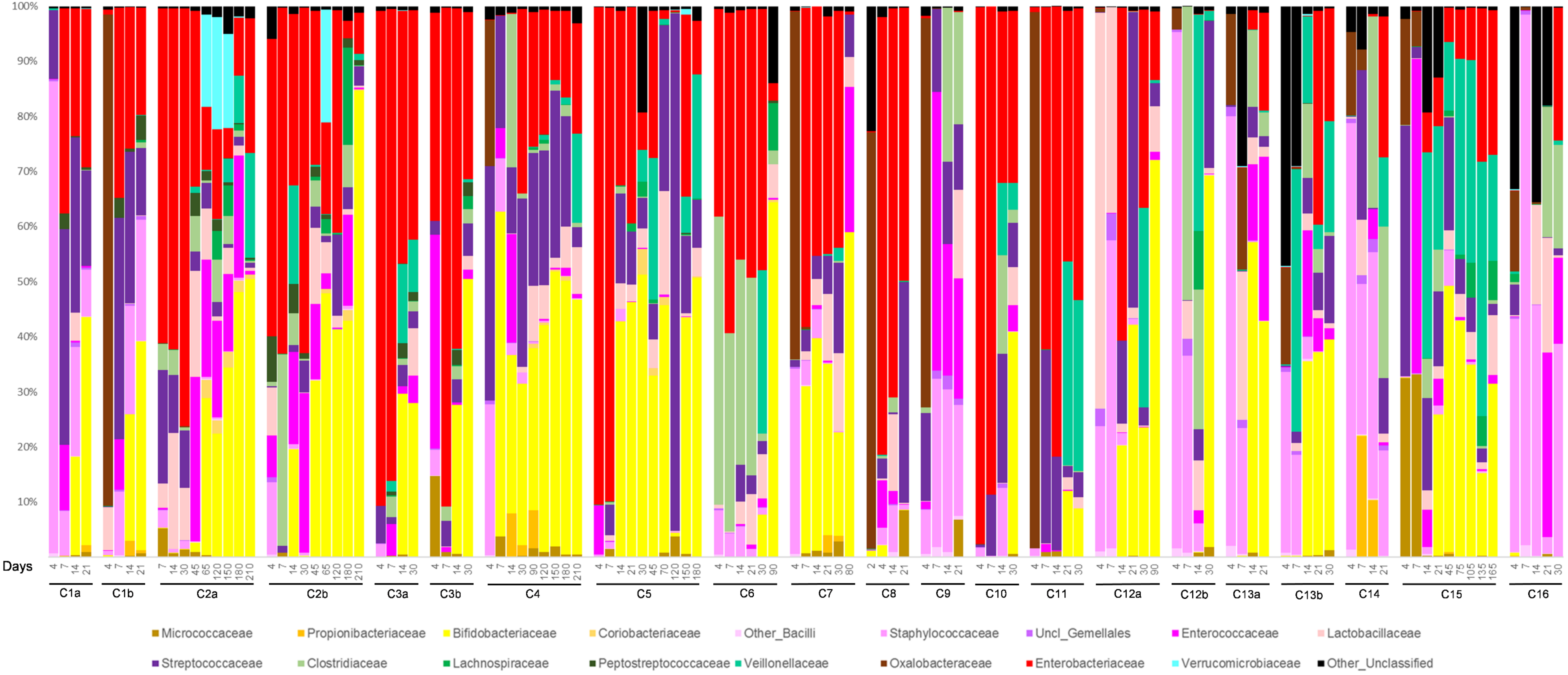

Supplementary Figure S5. Family-level relative abundance profiles of the oral microbiota of the enrolled 21 infants. Infant codes are reported at the bottom. Infants with the same number and differentiated by «a» and «b» letters are twins. Day of sampling is indicated for each sample. Bacterial families showing a minimum relative abundance of 1% in at least 4% of samples are plotted. Color legend is shown at the bottom.

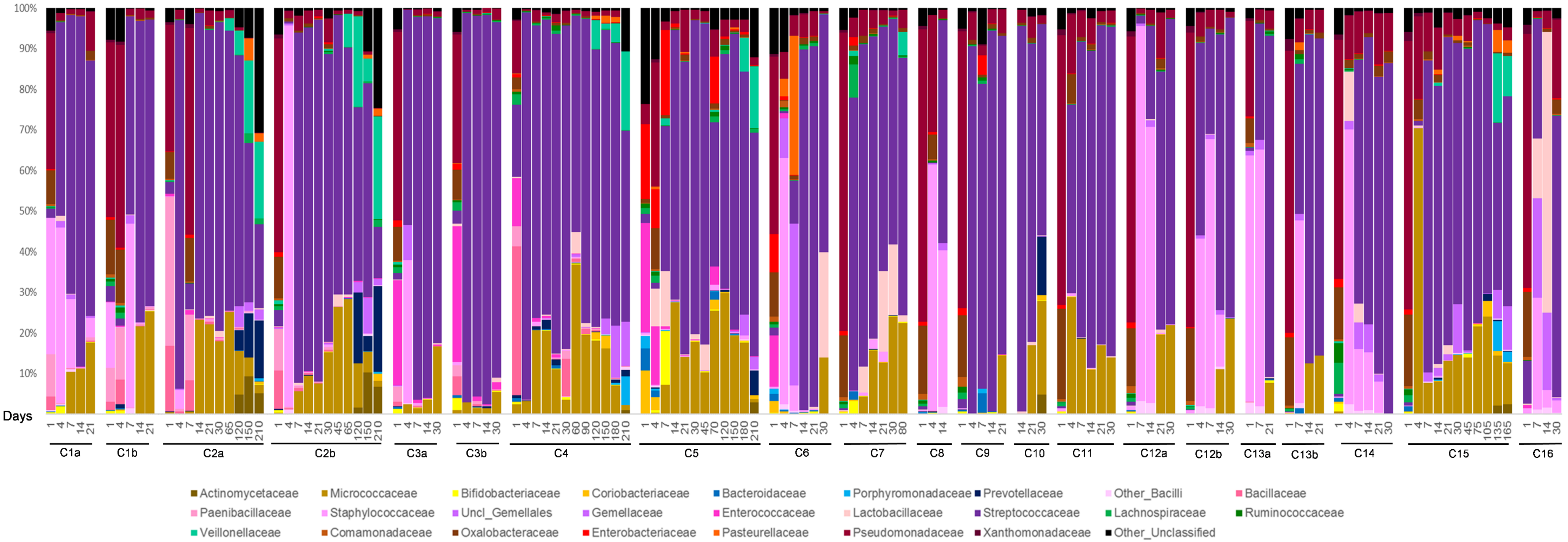

Supplementary Figure S6. Family-level relative abundance profiles of the mother's milk microbiota of 13 mothers. Mother codes are reported at the bottom. Day of sampling is indicated for each sample. Bacterial families showing a minimum relative abundance of 1% in at least 4% of samples are plotted. Color legend is shown at the bottom.

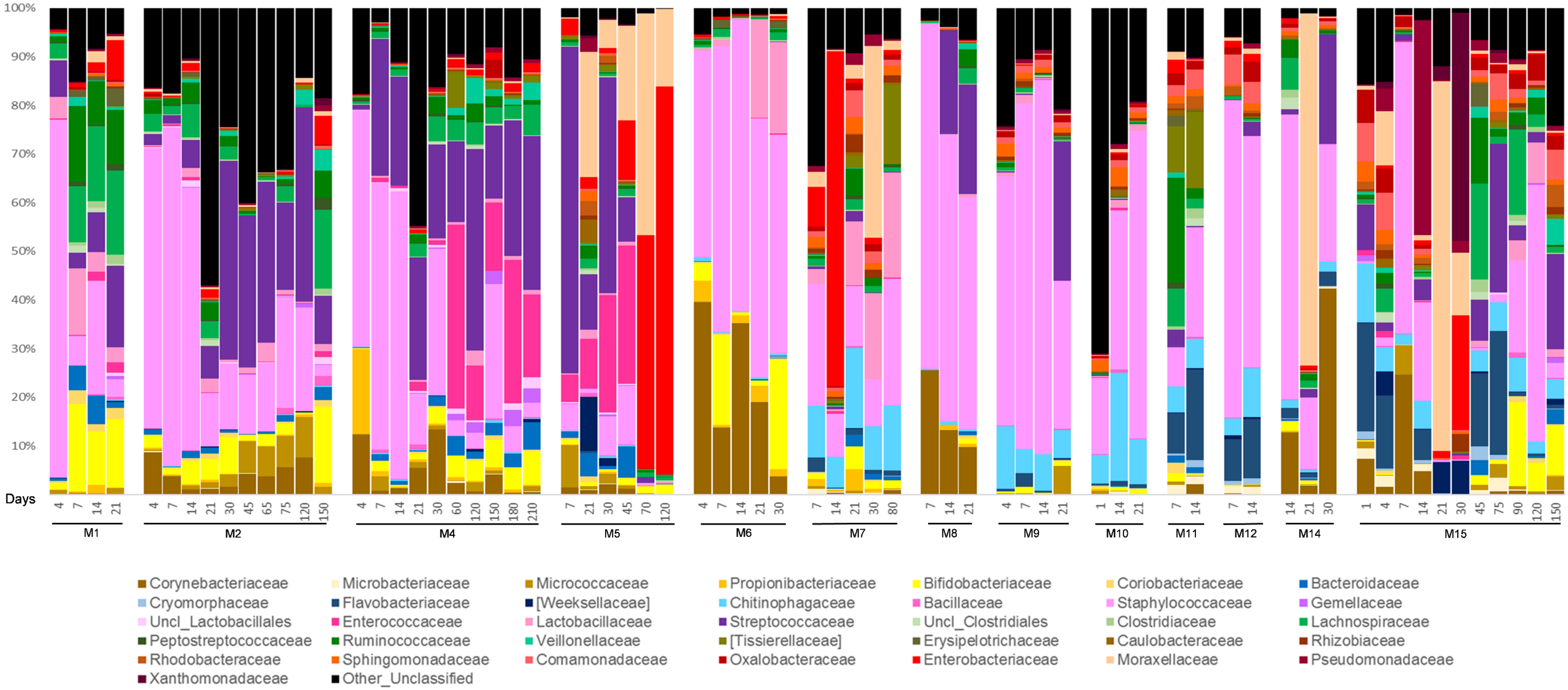

Supplementary Figure S7. The meconium microbiota. (A) Family-level relative abundance profiles. Bacterial families showing a minimum relative abundance of 1% in at least 4% of samples are plotted. Color legend is shown. (B) PCoA based on Bray-Curtis distances between genus-level profiles. First and second coordination axes, with percentages of plotted variation, are reported. Meconia taken from infants born by C-section or vaginal delivery are depicted as triangles or circles, respectively. In red, meconium samples taken from infants born after break of the membranes. Meconia were available only for 19 infants. Infants with the same number and differentiated by «a» and «b» letters are twins.

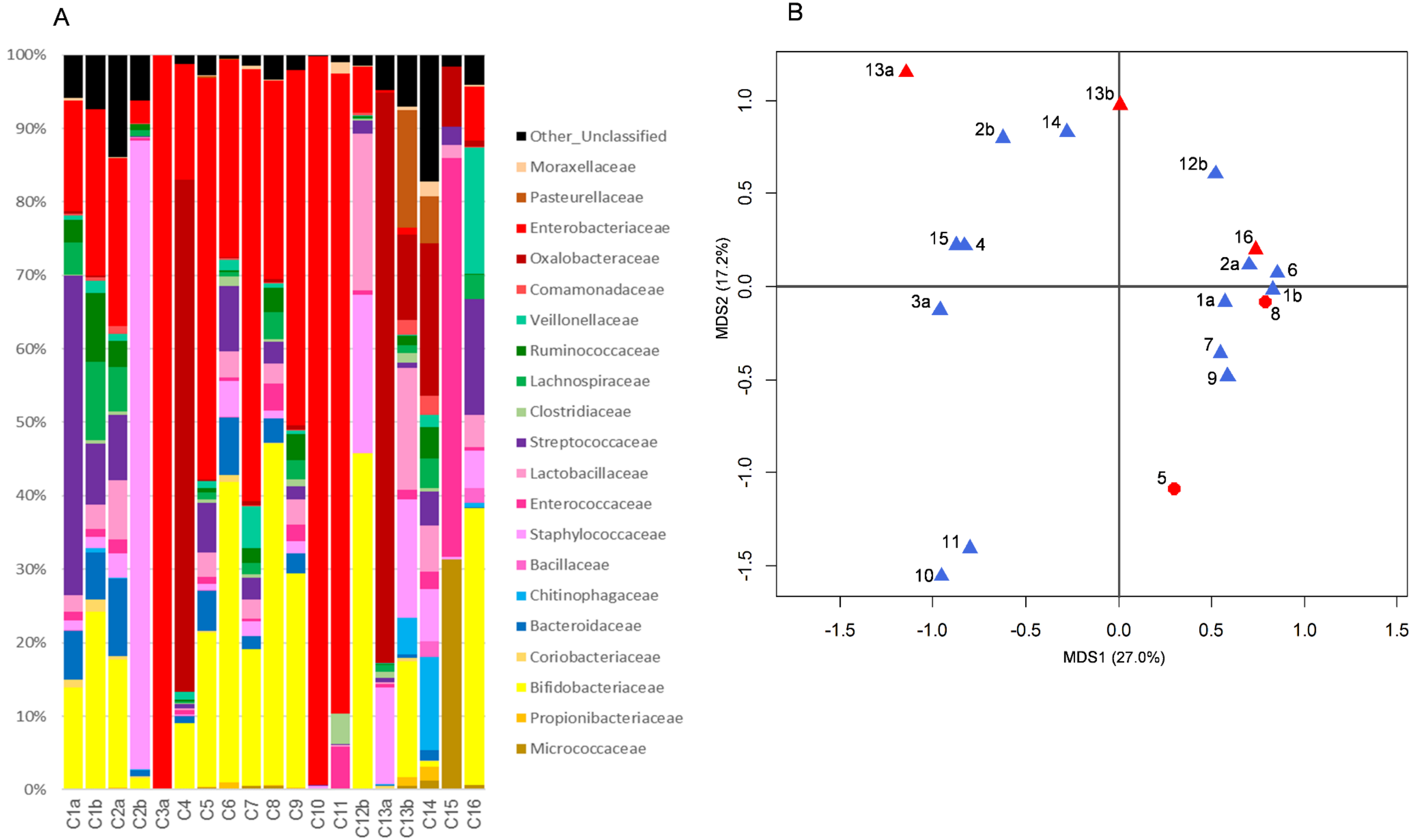

Supplementary Figure S8. PCoA based on Bray-Curtis distances between genus-level relative abundance profiles of the fecal and oral microbiota. Fecal (A) and oral (B) samples taken during infant feeding based on human milk only (mother or donor, purple), mixed milk (human and formula, orange) or formula only (green) are shown. Black dots identify samples taken when the infant was not fed for clinical reasons (see Supplementary Figure S1 and S2). First and second coordination axes, with percentages of plotted variation, are reported as in Fig. 1.

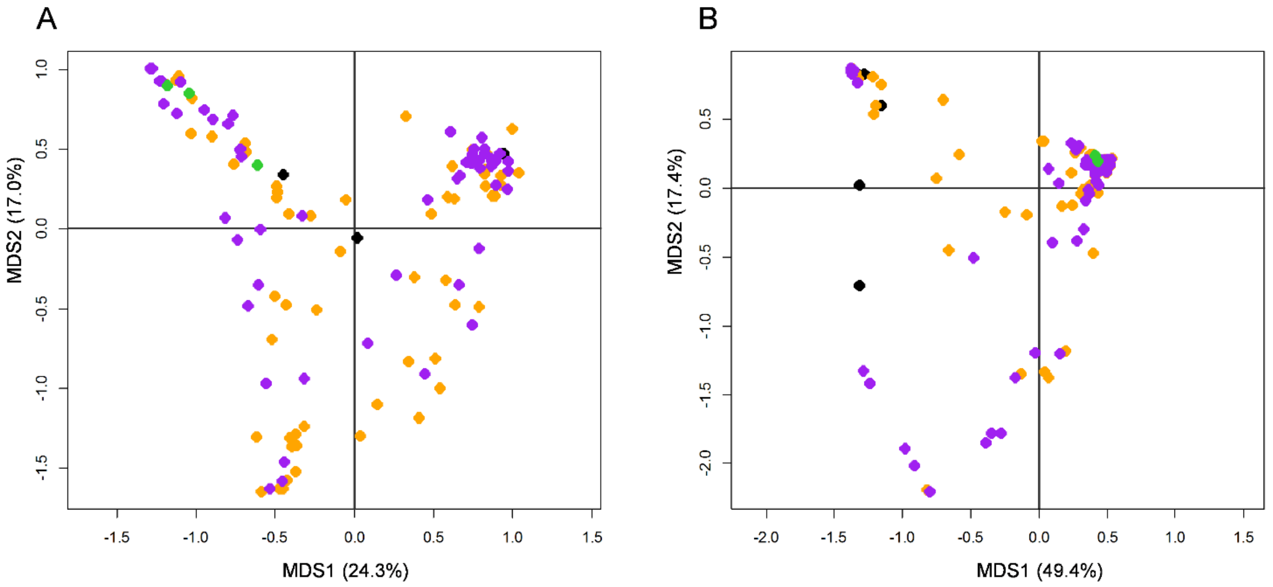

Supplementary Figure S9. Additional features of the three Milk Community Types (MCTs) (see Fig. 2). (A) PCoA based on Bray-Curtis distances between genus-level relative abundance microbiota profiles of mother's milk samples (as in Fig. 1C). Samples are depicted as dots colored according to the MCT into which they were clustered (pink, MCT1; gold, MCT2; blue, MCT3). First and second coordination axes are reported; percentages of variation in the dataset explained by each axis are reported. (B) Levels of alpha diversity calculated as number of observed OTUs for each MCT. Lines and stars below the plot indicate significant difference for pairwise comparison (post-hoc Tukey and Kramer test, P<0.05) (C) Percentages of milk samples taken before (white) or after (dark gray) actual breastfeeding started, assigned to each MCT.

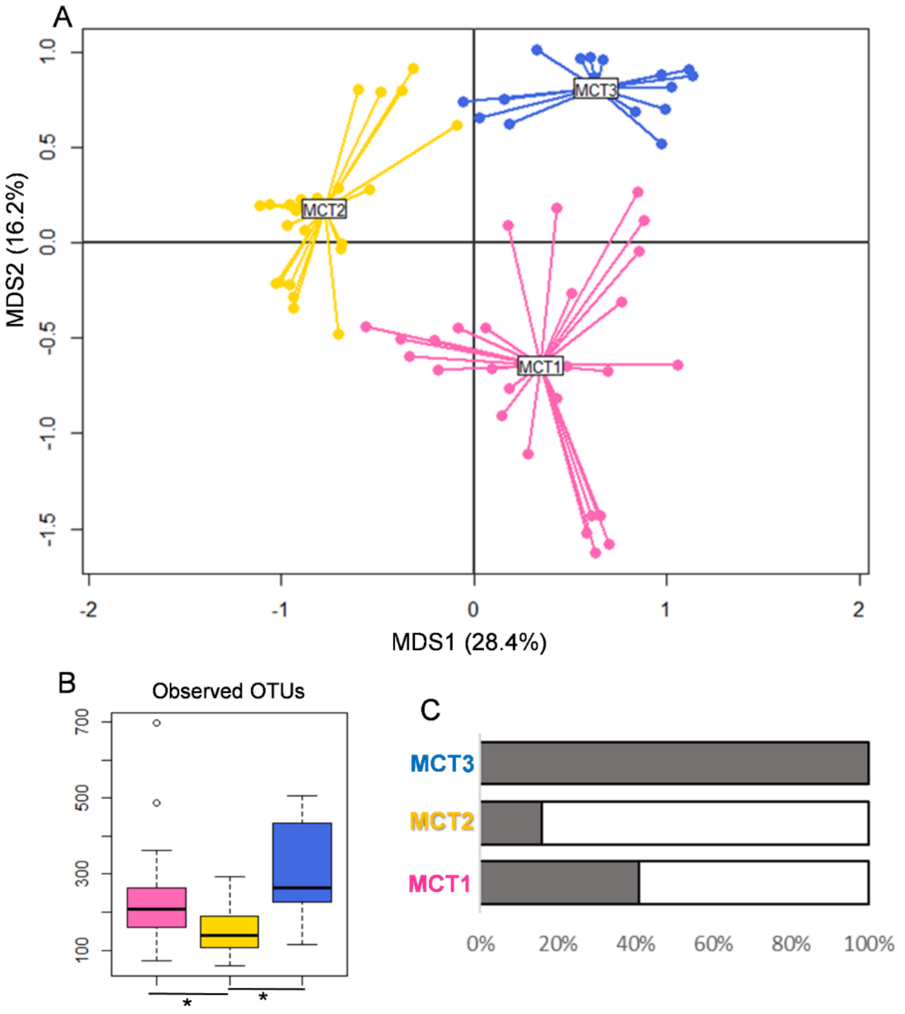

Supplementary Figure S10. The fecal and oral microbiota of infants in relation to the three Milk Community Types. PCoA based on Bray-Curtis distances between genus-level relative abundance profiles of fecal (A) and oral (B) samples, for which a corresponding milk sample (taken at the same time point) was available. Samples taken at time points in which the mother's milk harbored a MCT1 (pink), MCT2 (gold) or MCT3 (blue) microbiota are shown. First and second coordination axes, with percentages of plotted variation, are reported. Percentages of fecal (C) and oral (D) samples included in each MCT group that were taken during mixed feeding (MOM and formula, pink and green) or exclusive human milk feeding (MOM only, pink; mixed MOM and DHM, pink and blue). Black bars indicate the percentage of samples taken at time points at which the infant was not enterally fed because of clinical conditions.

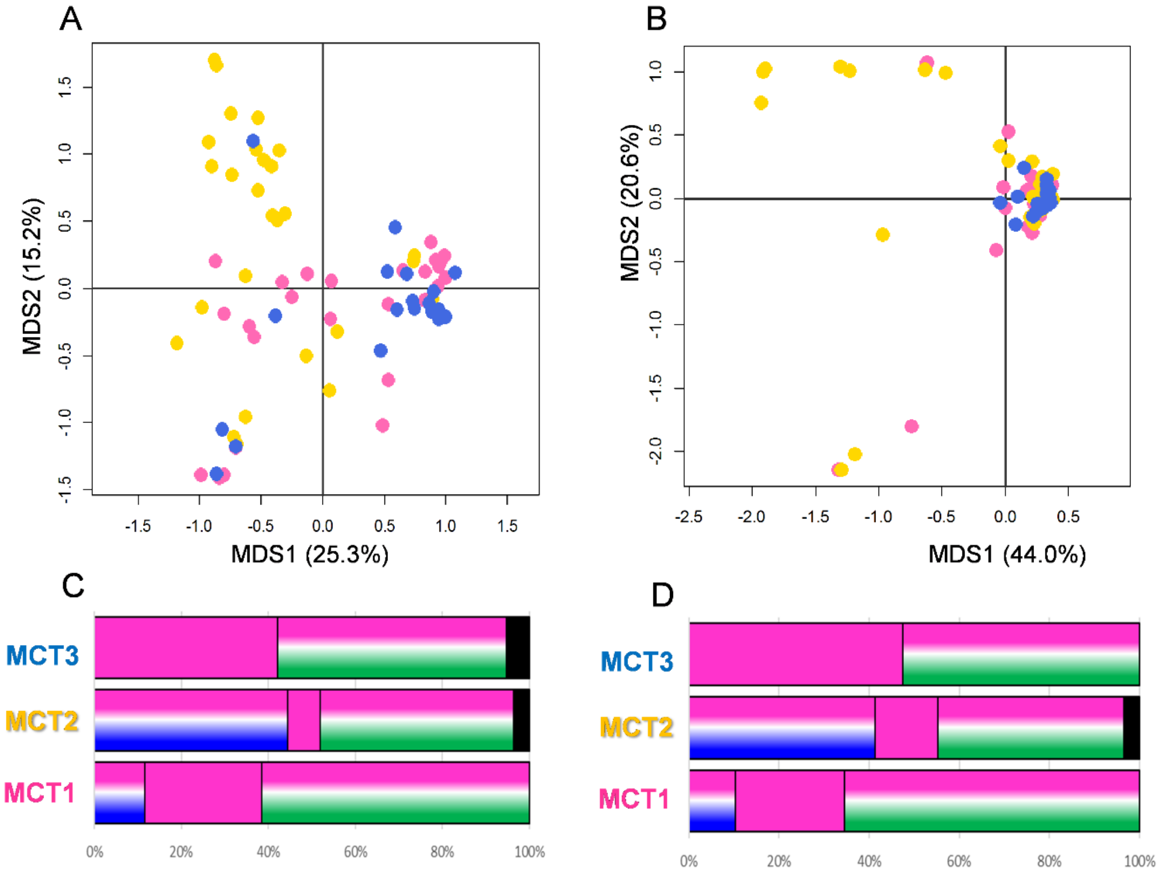

Supplementary Figure S11. Features of the infants' fecal and oral bacterial ecosystems associated with the three Milk Community Types. Boxplots of the relative abundances of bacterial genera for which a significant difference was observed (Kruskal-Wallis test, FDR corrected P<0.05) among fecal (A-D) or oral (E-G) samples taken at time points in which the corresponding milk samples harbored MCT1 (pink), MCT2 (gold) or MCT3 (blue). Lines and stars below each plot indicate significant differences for pairwise comparisons (post-hoc Tukey and Kramer test).

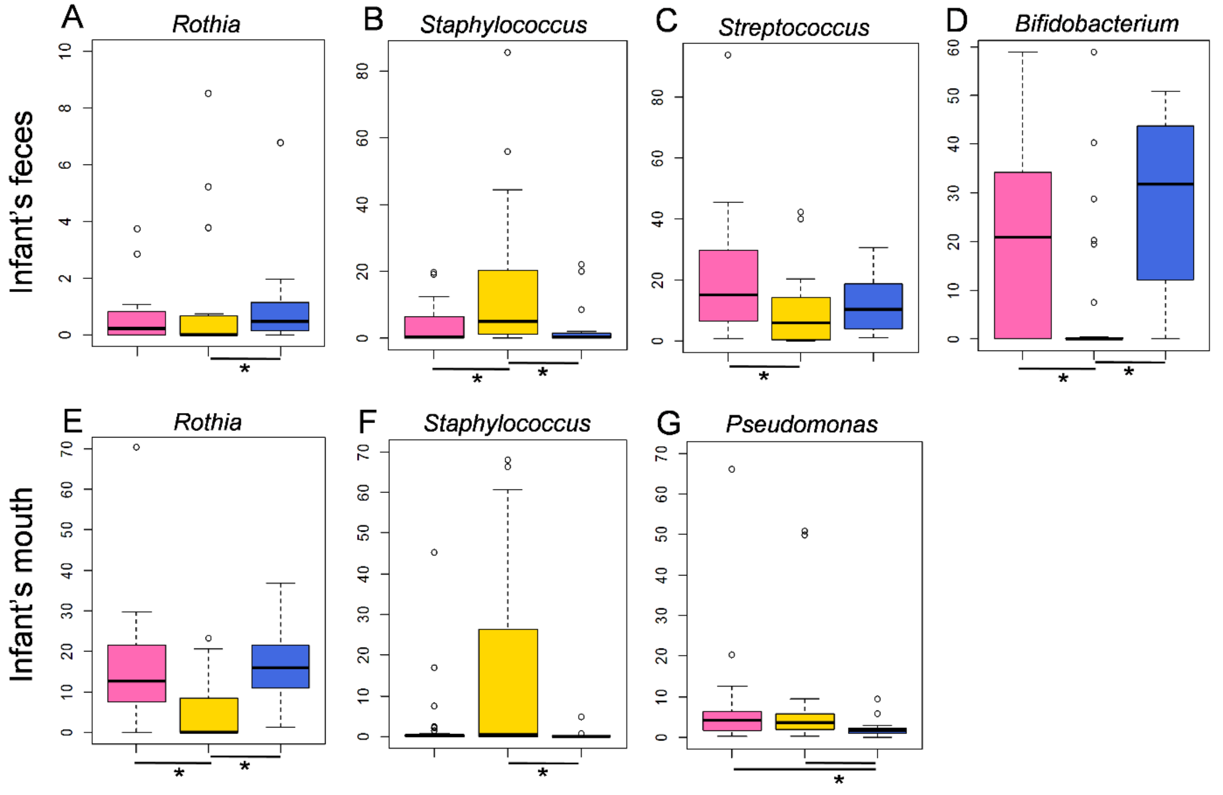

Supplementary Figure S12. Heat map showing the relative abundance of OTU518072, assigned to *Staphylococcus epidermidis*, in all samples taken from infants for which mother's milk samples were available (triads 3 and 13, and dyad 16 were excluded because milk samples had not been given by the mothers). For each available time point (expressed in days from birth above the heat map for each dyad/triad) the relative abundance of the OTU in all types of samples (M, milk; O, oral samples; F, feces) is shown in shades of green according to the color legend (right panel). The red line indicates the moment of infant latching to the breast.

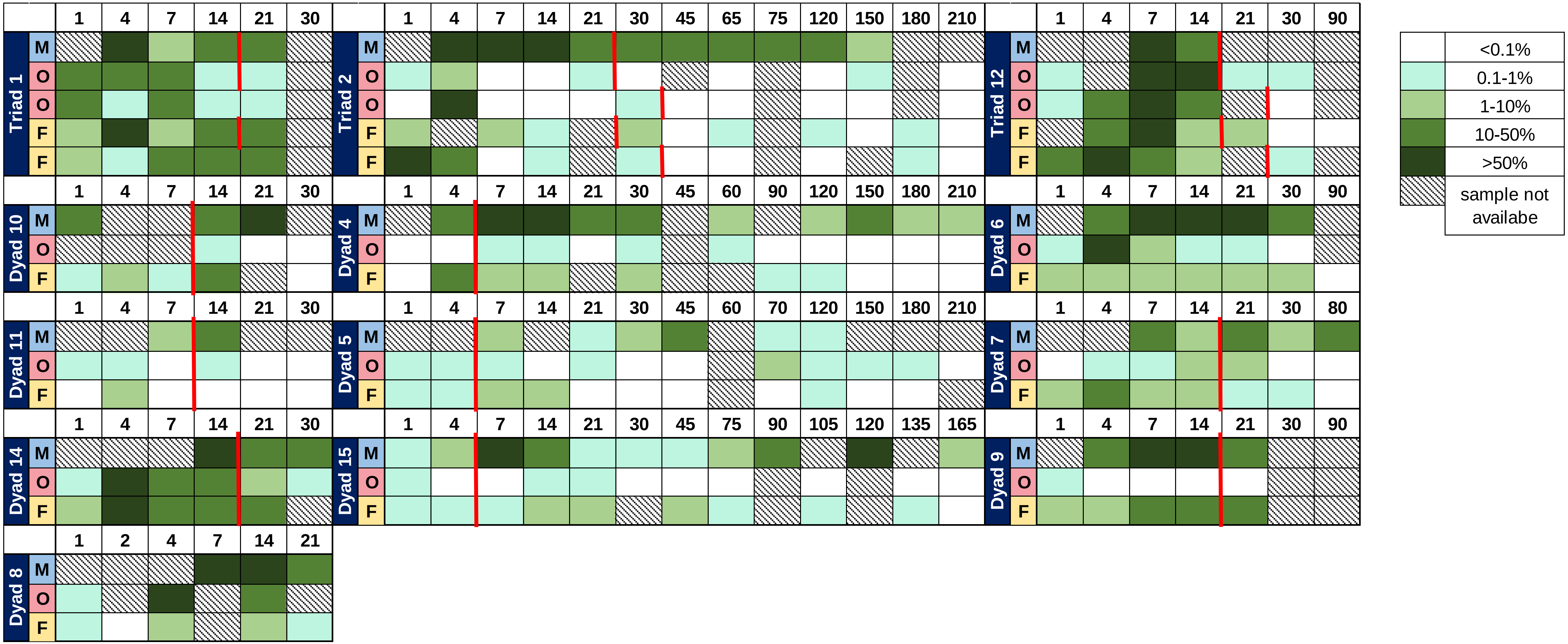

Supplementary Figure S13. Heat map showing the relative abundance of OTU537346, assigned to *Rothia mucilaginosa*, in all samples taken from infants for which mother's milk samples were available (triads 3 and 13, and dyad 16 were excluded because milk samples had not been given by the mothers). For each available time point (expressed in days from birth above the heat map for each dyad/triad) the relative abundance of the OTU in all types of samples (M, milk; O, oral samples; F, feces) is shown in shades of green according to the color legend (right panel). The red line indicates the moment of infant latching to the breast.

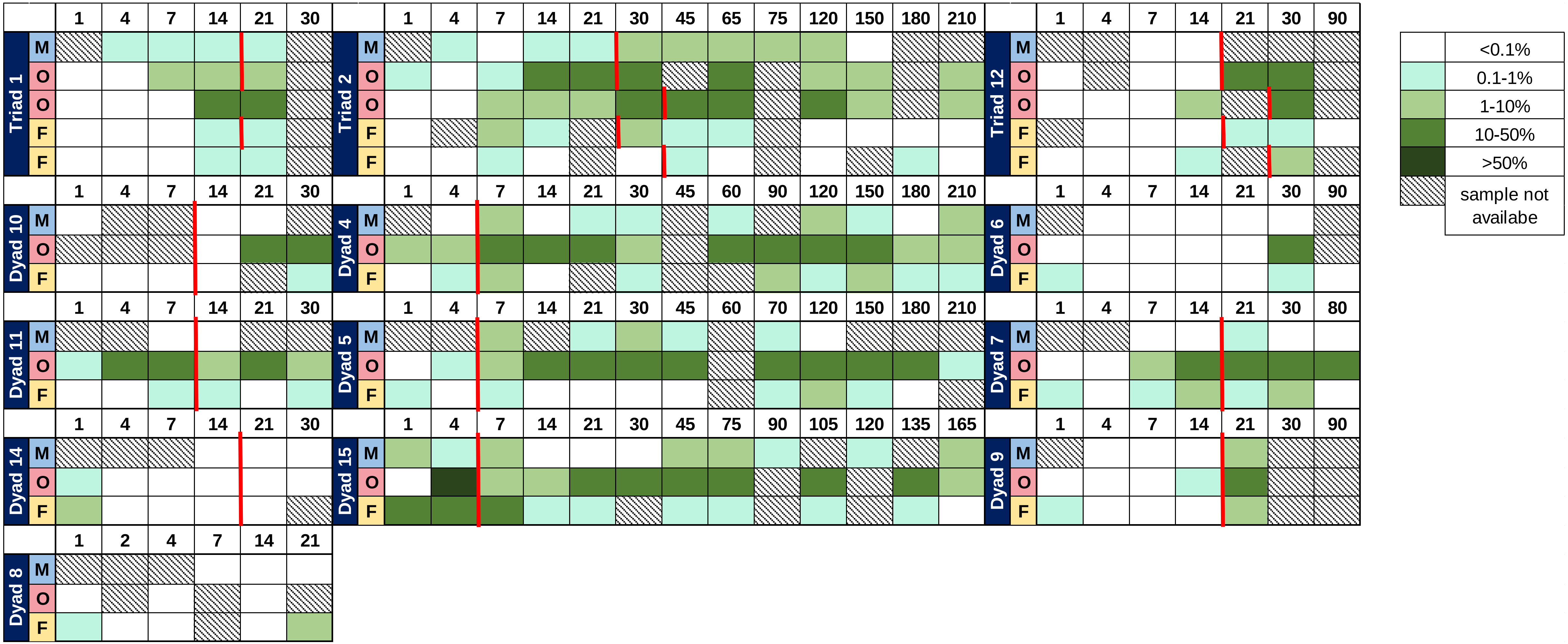

Supplementary Figure S14. Heat map showing the relative abundance of OTU30207, assigned to *Pseudomonas* spp., in all samples taken from infants for which mother's milk samples were available (triads 3 and 13, and dyad 16 were excluded because milk samples had not been given by the mothers). For each available time point (expressed in days from birth above the heat map for each dyad/triad) the relative abundance of the OTU in all types of samples (M, milk; O, oral samples; F, feces) is shown in shades of green according to the color legend (right panel). The red line indicates the moment of infant latching to the breast.

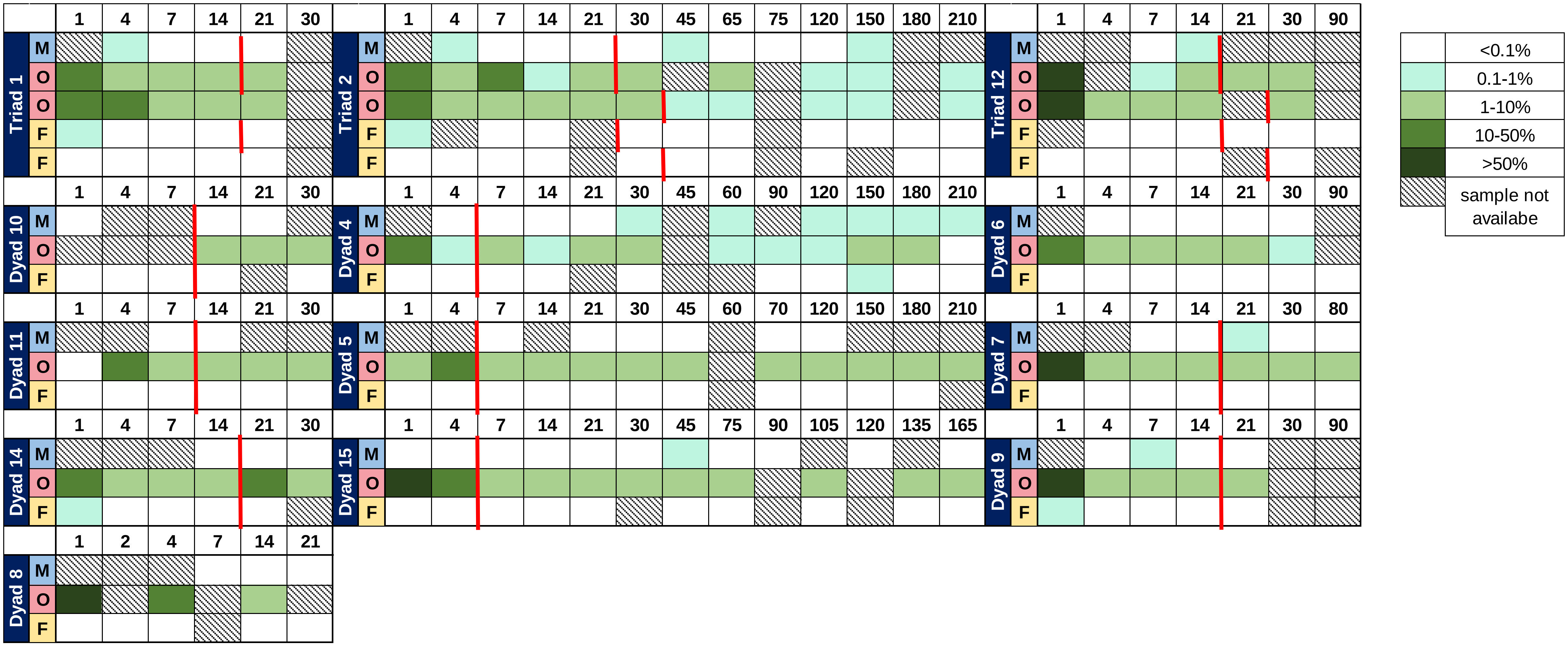

Supplementary Figure S15. Heat maps showing the relative abundance of OTU94700 (A) and OTU420746 (B), assigned to *Bifidobacterium longum* and *Bifidobacterium breve*, respectively, in all samples taken from infants for which mother's milk samples were available (triads 3 and 13, and dyad 16 were excluded because milk samples had not been given by the mothers). For each available time point (expressed in days from birth above the heat maps for each dyad/triad) the relative abundance of the OTU in all types of samples (M, milk; O, oral samples; F, feces) is shown in shades of green according to the color legend (see Figure S11). The red line indicates the moment of infant latching to the breast.

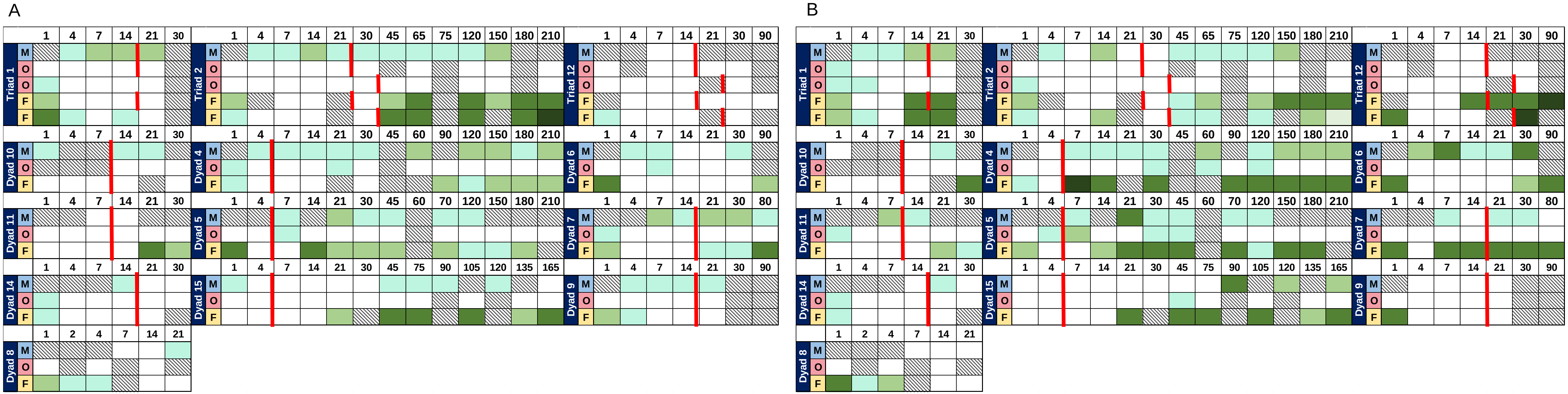

Supplement: Supplementary file 1 [file Data_Sheet_1.pdf]
